# Metagenomic Characterization of *Candidatus* Smithella cisternae Strain M82_1, a Syntrophic Alkane-Degrading Bacteria, Enriched from the Shengli Oil Field

**DOI:** 10.1264/jsme2.ME17022

**Published:** 2017-09-27

**Authors:** Qian-Shan Qin, Ding-Shan Feng, Peng-Fei Liu, Qiao He, Xia Li, Ai-Ming Liu, Hui Zhang, Guo-Quan Hu, Lei Cheng

**Affiliations:** 1 Key Laboratory of Development and Application of Rural Renewable Energy, Biogas Institute of Ministry of Agriculture Chengdu 610041 P. R. China; 2 Anhui Normal University Wuhu 241000 P. R. China

**Keywords:** hydrocarbon degradation, metabolic traits, metagenomics, methanogenesis, *Syntrophaceae*

## Abstract

The methanogenic degradation of hydrocarbons plays an important role in hydrocarbon-contaminated environments in the absence of an external electron acceptor. Members of *Syntrophaceae* sublineages were previously reported to be responsible for syntrophic alkane degradation. However, limited information is currently available on their physiological capabilities in nature because it is very challenging to cultivate these as-yet uncultured microbes. We herein performed metagenomic sequencing of the methanogenic hexadecane-degrading culture M82 and recovered a nearly complete genome (2.75 Mb, estimated completeness ≥97%) belonging to *Syntrophaceae* sublineage II. The assembly genome was tentatively named “*Candidatus* Smithella cisternae strain M82_1”. Genes encoding alkylsuccinate synthase for alkane activation were identified, suggesting that this organism is capable of oxidizing alkanes through fumarate addition. This capability was further supported by the detection of methyl pentadecyl succinic acid and methyl tetradecyl succinic acid in cultures amended with hexadecane and pentadecane, respectively. Genes encoding enzymes for the β-oxidation of long-chain fatty acids and butyrate were also identified. The electron transfer flavoprotein/DUF224 complex is presumed to link electron flow from acyl-CoA dehydrogenase to a membrane hydrogenase or formate dehydrogenase. Although no indications of Rnf complexes were detected, genes encoding electron-confurcating hydrogenase and formate dehydrogenase were proposed to couple the thermodynamically favorable oxidation of ferredoxin to generate H_2_ and formate from NADH. Strain M82_1 synthesized ATP from acetyl-CoA by substrate-level phosphorylation or F_1_F_0_-ATP synthases. These results provide an insight into the potential metabolic traits and ecophysiological roles of the syntrophic alkane degrader *Syntrophaceae*.

Alkanes consist of hydrogen and carbon atoms only, all their bonds are single bonds, and these molecules are major constituents of natural gas and petroleum (59). Alkanes may also be generated by some microorganisms, plants, and animals for specific or unknown biological activities (66). Alkanes are relatively inert, which make them unreactive to most chemical transformations (40), whereas the biodegradation of alkanes appears to widely occur under environmental conditions, and aerobic microorganisms that degrade alkanes have been known for more than a century and studied in detail (62). Alkane-activating enzymes (monooxygenases) overcome the low chemical reactivity of hydrocarbons by inserting a reactive oxygen species (62). The resulting alcohols are further oxidized to their corresponding aldehydes, and are ultimately converted into fatty acids (62).

Although it was previously unclear whether the microbial degradation process occurs in the absence of oxygen, Aeckersberg and co-workers succeeded in isolating and characterizing anaerobic bacteria that degrade alkanes with sulfate as a terminal electron acceptor in 1991 (1). The growth profiles of anaerobic hydrocarbon-degrading microorganisms with diverse electron acceptors have frequently been investigated (88). Under conditions of external electron acceptor limitations, the anaerobic conversion of hexadecane to methane was demonstrated in an enriched microcosm in which *Syntrophus* spp. were detected as the dominant bacterial phylotypes (92). Since these *Syntrophus* spp. are phylogenetically distant from known *Syntrophus* isolates (*i.e.*, *Syntrophus aciditrophicus*, *S. buswellii*, and *S. gentianae*), the genus *Smithella* was proposed with *Smithella propionica* as a cultured representative (45). Methanogenic alkane degradation is also a common process in subsurface degraded oil reservoirs (36). Laboratory incubation experiments revealed that a *Syntrophus* sp. was dominant in the microcosms (36). This bacterial phylotype was further proposed to be responsible for alkane degradation by a combined analysis of the 16S rRNA gene of individual microbial phylotypes and methane production over time (30). By using DNA-stable isotope probing with UL-^13^C-hexadecane, we previously demonstrated that another uncultured *Syntrophaceae* species represents a novel syntrophic alkane degrader (13).

Studies on syntrophic alkane-degrading microbial communities have provided essential insights into the ecophysiology of *Syntrophaceae*. However, difficulties have been reported in further investigations because efforts to isolate these *Syntrophaceae*-related organisms have been unsuccessful. Due to the absence of cultured representatives of syntrophic alkane-degrading bacteria, single-cell genomics and assemblies of metagenomes provide new opportunities to investigate the genetic potential of uncultured *Syntrophaceae* from complex microbial communities (24, 25, 79, 87). Tan *et al.* (79) applied a metagenomic approach to reconstruct syntrophic short-chain alkane degraders from methanogenic cultures enriched from oil sands mature fine tailings, but only obtained a partial genome of a syntrophic alkane degrader. However, putative alkylsuccinate synthase gene (*ass*A) analogues were detected in the metagenome, suggesting that hydrocarbon degradation may be initiated through the addition of fumarate. Embree *et al.* (24) applied a single-cell genome sequencing technique to assemble a *Smithella* draft genome from a methanogenic hexadecane-degrading culture. They also identified strongly expressed genes associated with radical-activating enzymes and hypothetical proteins through a metatranscriptomic analysis, but failed to assign them to *ass*A genes (24). It soon became clear that *Smithella* may degrade alkanes by the addition of fumarate under methanogenic conditions (81) according to a re-analysis of omics data reported by Embree *et al.* (24). Wawrik *et al.* (87) performed metagenomics and RT-PCR analyses and revealed that *Smithella* spp. activated paraffin degradation via “fumarate addition”.

*Syntrophaceae* members have been repeatedly detected in methanogenic alkane-degrading cultures enriched from geographically distant sites (10, 14, 28, 72, 73, 85). These *Syntrophaceae* related-clones were primarily divided into three sublineages, which may represent three genera with different ecophysiological properties (20). To date, three draft genomes of uncultured syntrophic alkane degraders belonging to sublineage I have been reported (24, 80); however, only one sublineage II genome has been published (87). In the present study, we characterized the microbial diversity of the methanogenic alkane-degrading culture M82 using 454 pyrosequencing of the 16S rRNA gene, and assembled a nearly complete genome using metagenome construction and binning technology. We also identified metabolites generated from alkane degradation using gas chromatography-mass spectrometry (GC-MS). A draft genome of “*Candidatus* Smithella cisternae” strain M82_1 was constructed from the metagenome of the sublineage II *Syntrophaceae*-dominated culture M82. Additionally, the genetic potential of strain M82_1 was characterized. Based on these results and those of an intermediate analysis, alkane activation through fumarate addition was proposed.

## Materials and Methods

### Methanogenic incubation

The methanogenic hexadecane-degrading culture M82 was enriched from the oily sludge-contaminated sediment of the Shengli oil field, which is China’s third largest oil field and located in eastern China (13). The culture was incubated at 35°C as described previously (13), and cultures grown to the late-exponential phase were harvested for genomic DNA (gDNA) extraction. M82 was subsequently transferred to freshwater media amended with hexadecane (100 μL, Sigma-Aldrich, St. Louis, MO, USA) or pentadecane (100 μL, Sigma-Aldrich), and then incubated statically at 35°C in the dark. After reaching the mid-exponential growth phase, both cultures were collected for a metabolite analysis. A control culture without alkane addition was also incubated and prepared for the metabolite analysis.

### Metabolite analysis

Exponential-phase cultures (80–100 mL) amended with hexadecane or pentadecane were transferred to clean glass vials under the protection of N_2_ and treated with NaOH (pH>12) for 30 min. Control cultures without hexadecane and pentadecane addition were also collected at the same time. The cultures were then preserved at pH<2 by the addition of 6 M HCl prior to extraction for putative metabolites. Samples were extracted three times with 70 mL of ethyl acetate. The extracts were dried over anhydrous Na_2_SO_4_ and concentrated to a volume of 30 to 50 μL by rotary evaporation under a flow of N_2_. The organic extracts were then allowed to react with 400 μL of N,O-bis(trimethylsilyl)trifluoroacetamide (BSTFA, Sigma-Aldrich) and were incubated at 65°C for 45 min. Trimethylsilyl (TMS) derivatives were analyzed using GC-MS (Agilent 7890A-5975C, Agilent Technologies, Santa Clara, CA, USA) with a DB-5MS capillary column (30 m×0.25 mm×0.25 μm). Oven temperature remained at 40°C for 2 min, was increased to 270°C at a rate of 4°C per min, and was then held at 270°C for 10 min. The mass transfer line temperature was 280°C. Mass spectral data were generated using a mass spectrometer (Agilent 5975C, Agilent Technologies) at an electron energy of 70 eV in the SCAN/SIM mode. These data were initially used to search the NIST11 mass spectral library in order to obtain a reference. Mass spectral metabolites were also referenced to previously reported fragments, which represented either fumarate addition (8, 16, 17, 39) or the carboxylation (9, 76) of alkane degradation.

### Sample collection and DNA extraction

gDNA was extracted from 8- to 12-mL cultures using a modified beating method (15) without bead addition, and the beating speed was changed to 4.0 ms^−1^. gDNA was purified through agarose gel electrophoresis, and DNA concentrations were measured using a NanoVue spectrophotometer (GE Healthcare, Chicago, IL, USA). Purified gDNA was stored at –80°C until used.

### Terminal Restriction Fragment Length Polymorphism (T-RFLP) analysis

Methanogenic cultures amended with pentadecane and hexadecane after a 298-d incubation were also collected for gDNA extraction using a bead-beating method. The T-RFLP analysis for archaeal and bacterial profiles was performed as described previously (14, 15). Briefly, *Taq* I and *Msp* I were applied for archaeal and bacterial DNA digestion, respectively (14, 15).

### 454 pyrosequencing of the 16S rRNA gene (16S pyrotag)

Fragments of bacterial and archaeal 16S rRNA genes were amplified using the primers B27f/B533r (B27f: 5′-AGAGTTTGATCMT GGCTCAG-3′, B533r: 5′-TTACCGCGGCTGCTGGCAC-3′) and A344f/A915r (A344F: 5′-ACGGGGYGCAGCAGGCGCGA-3′, A915R: 5′-GTGCTCCCCCGCCAATTCCT-3′), respectively, with Roche 454 sequencing adapters (Table S1) (44). The first PCR reaction (20 μL) contained 2.5 U of Ex *Taq* DNA polymerase (Takara, Otsu, Japan), 2 μL of 10×buffer, 1.6 μL of dNTPs, 0.4 μL of each primer, 1 μL of bovine serum albumin (BSA), 1 μL of template DNA, and 13.1 μL of water. The PCR conditions used for bacterial amplification were as follows: 5 min at 95°C; 26 cycles of denaturation at 94°C for 30 s, annealing at 56°C for 30 s, and primer extension at 72°C for 2 min and a final extension at 72°C for 10 min. PCR conditions for archaea were as follows: 94°C for 4 min, 25 cycles of 94°C for 1 min, 52°C for 1 min, and 72°C for 2 min, and a final extension at 72°C for 7 min. Purified PCR products were quantified by fluorimetry with a Qubit^®^ dsDNA HS Assay kit (Invitrogen, Waltham, MA, USA) and were then pooled and sequenced using a GS FLX Titanium LV emPCR Kit on a GS FLX+ Instrument (Roche, Indianapolis, IN, USA) according to the manufacturer’s instructions.

### 16S pyrotag data analysis

Raw pyrosequencing sequences were analyzed using Mothur v.1.31.2, mainly according to the standard operating procedure (67). Briefly, barcode and primer sequences were both trimmed, and sequences with homopolymers exceeding 8 bp and shorter than 200 bp were removed, allowing for 1 mismatch to the barcode and 2 mismatches to the primer. Chimeras were identified using the chimera.uchime command in Mothur. After denoising, sequences were clustered into operational taxonomic units (OTUs) at a 97% sequence similarity threshold, and coverage was calculated according to Good’s formula (29). Representative clones from each OTU were assigned to taxonomic ranks using the RDP classifier (RDP 11.1 database) with a confidence threshold of 50% (86). Raw 454 pyrosequencing reads were submitted to the NCBI Sequencing Read Archive (SRA) database under the following accession numbers: SRR1257359 (bacteria) and SRR1258059 (archaea).

### Metagenomic sequencing and assembly

A paired-end sequencing library with a size of 160±8 bp was constructed from high-molecular-weight DNA (*ca.* 23 kb, 8–10 μg) and was sequenced using the Illumina HiSeq 2000 system at BGI-Shenzhen, generating *ca.* 41 Gb of raw data. Sequence quality trimming was performed by Trimmomatic with a minimum quality score of 20 and minimum sequence length of 36 bp (6). *De novo* assembly was conducted using IDBA-UD (v. 1.1.1) with kmers of 20 (min) and 90 (max) and a min-contig size of 200 bp (57). The relative abundance levels of scaffolds were calculated according to reads mapped to each scaffold by Bowtie2 (42) and SAMtools (43). The G+C content and tetranucleotide frequency (TNF) for each scaffold were calculated according to a previous study (2). The taxonomic assignments of metagenomic scaffolds were obtained through sequence homology (33) and composition methods (56).

### Binning and annotation of the *Candidatus* Smithella cisternae strain M82_1 genome

Given the dominant abundance of the *Candidatus* Smithella cisternae lineage in the M82 culture, the primary bin was generated according to scaffold coverage and the G+C content (2), while scaffolds assigned to the archaeal domain were excluded. Reads in the primary bin were reassembled using Velvet (kmer length=81 bp) (93), in which scaffolds with low abundance (<300) were removed. Open reading frames (ORFs) in the metagenomic scaffolds were predicted using Prodigal (34). In order to estimate genome completeness, bacterial essential single-copy genes in the primary bin were retrieved using HMMER 3.0 (26), and amino acid sequences were analyzed using BLASTP against the RefSeq protein database with a maximum e value cut-off of 1e–5 according to Albertsen *et al.* (2). rRNA and tRNA genes were predicted by RNAmmer (41) and tRNAscan-SE (64), respectively. Non-coding RNA genes were annotated using Rfam (31). Clustered regularly interspaced short palindromic repeats (CRISPRs) were identified and analyzed using the on-line CRISPRFinder (32). All ORFs were automatically annotated using the RAST server (4). Gene products were classified into functional categories by performing a BLAST search against the Cluster of Orthologous Groups (COG) database (3). Proteins with transmembrane helices were identified using the TMHMM Server v. 2.0 (50), and twin-arginine translocation (Tat) motifs in the N terminus were identified to predict protein localization to the cell membrane using the Tat P 1.0 Server (5). Illumina raw sequence reads were submitted to the SRA database under accession number SRP072262, and binned draft genome sequences were submitted to NCBI under accession numbers: MAEO01000001–MAEO01000236.

## Results and Discussion

### General characteristics of the community metagenome

**Fig. 1:**
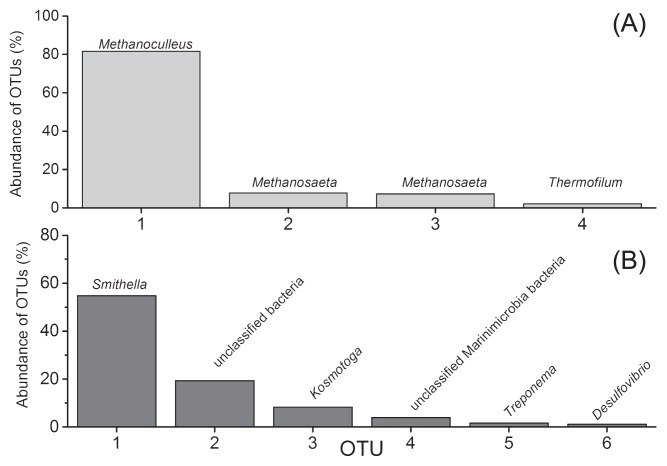
Archaeal (A) and bacterial (B) community compositions analyzed via 454 pyrosequencing. OTUs with an abundance lower than 1% in each domain were not shown.

A total of 2163 and 54215 sequences for the archaeal and bacterial domains, respectively, were used for a microbial composition analysis (Tables S2 and S3). These sequences were clustered into OTUs based on 97% sequence similarity, and the values of Good’s coverage in both libraries were more than 99% (Table S2 and S3). The dominant species in the archaeal domain mainly belonged to *Methanoculleus* (82% of total archaeal sequences), *Methanosaeta* (15%, two OTUs), and *Thermofilum* (2%) (Fig. 1A). The bacterial phylotypes at the OTU levels were mainly divided into *Smithella* (55% of bacterial sequences), unclassified *Parcubacteria* bacteria (19%), *Kosmotoga* (8%), unclassified *Marinimicrobia* bacteria (4%), *Treponema* (2%), and *Desulfovibrio* (1%) (Fig. 1B). The microbial community structure was similar to our previous analysis (14). The type sequence (IDP5YSY02DSHBX) of the predominant OTU exhibited identical sequence similarity to clone HB1_11, which representing the *Syntrophaceae* key player responsible for hexadecane degradation, and shared 95.6% sequence similarity to *S. propionica* (AF126282) (13, 30). This novel hexadecane degrader (*Smithella* sp.) is affiliated with *Syntrophaceae* sublineage II, which has been proposed as a candidate species for syntrophic alkane degradation bacteria (20). However, the metabolic potential of alkane-degrading bacteria in *Syntrophaceae* sublineage II (20) has not yet been examined in detail.

**Fig. 2:**
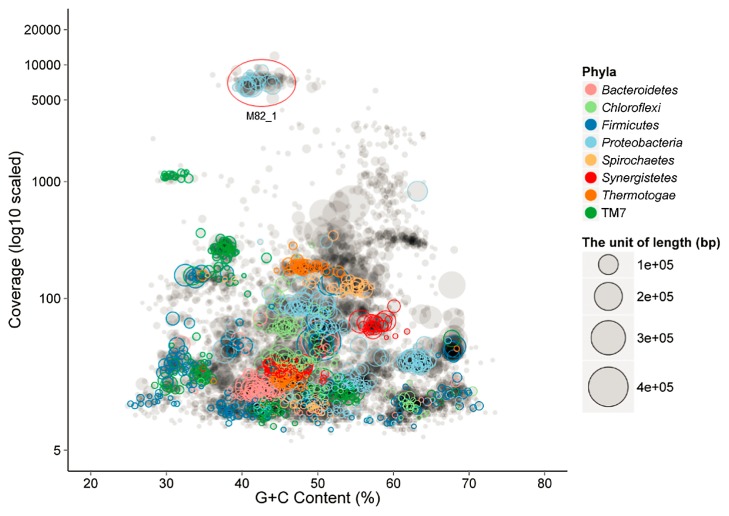
Metagenomic scaffolds plotted by average coverage and the G+C content (%). Each circle represents a scaffold with a size proportional to its length and colored by phylum. Clusters of scaffolds represent putative genome bins.

A total of 41.5 Gb of sequence data was obtained from this M82 culture using the Illumina HiSeq platforms, yielding a 208-Mbp metagenome containing 144,759 scaffolds between 200 and 482,817 bp (N_50_ size=4,619 bp, Table 1). PhyloPythia assigned approximately 55% of the sequence fragments to the phylum *Proteobacteria* (46), and binning of these scaffolds into population genomes was facilitated by plotting coverage and the G+C content (Fig. 2). Scaffolds with high coverage (>3,000) and an average G+C content of 43% were binned into one cluster (Fig. 2). The phylogenetic analysis revealed that the 16S rRNA gene retrieved from this cluster exhibited 96% sequence similarity to *S. propionica*, and shared nearly identical sequence similarity (99.7%) with clone HB1_11, which represents a syntrophic hexadecane degrader through the DNA stable isotope probing technique (13). These results indicated that this bin most likely belonged to the uncultured syntrophic hexadecane degrader *Smithella*. Furthermore, low quality sequences, *e.g.*, low-abundant scaffolds less than 300 or sequences belonging to the archaeal domain, were removed after the reassembly of reads in the primary bin. A principal component analysis (PCA) of the TNF of the post-binning sequences showed that *Smithella* scaffolds were tightly clustered (Fig. S1). Previous studies reported that the abundance of single-copy marker genes (the total number was 107 in 95% of all sequenced bacteria) may be used to assess genome completeness (2, 22). In the present study, 104 unique single-copy genes were identified in this bin, indicating genome completeness greater than 97% (Table S4). The draft genome of this bin consisted of 236 scaffolds, had a total size of 2,753,163 bp with a G+C content of 42.8%, and harbored 2815 ORFs (Table 1).

### Comparison with other *Smithella* genomes

By comparing the 2815 ORFs of M82_1 to publicly reported genomes in the NCBI non-redundant (NCBI-nr) database using the best BLAST hit with a score of E<e–5, we found that the closet associations were with syntrophic microorganisms (Fig. S2), such as *Smithella* sp. SDB (1,420 best-hint genes), *Smithella* sp. ME-1 (287), *Smithella* sp. SCADC (204), and *Smithella* sp. SC_K08D17 (165). A total of 1,937 ORFs (69%) were categorized into 21 functional COGs representing energy production and conversion (subgroup C, 174 ORFs), cell wall/membrane/envelope biogenesis (subgroup M, 167), and replication recombination and repair (subgroup L, 161; Tables 1 and S5).

### Central metabolism

**Fig. 3:**
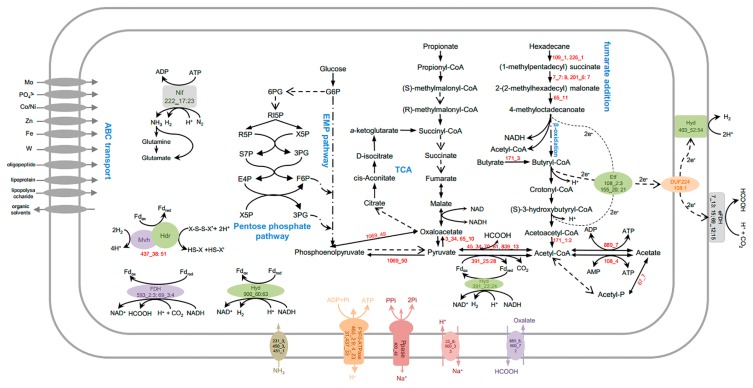
Overview of the metabolism of *Candidatus* Smithella cisternae. Abbreviations: ribulose-5-P: RI5P; ribose-5-P: R5P; sedoheptulose-7-P: S7P; glyceraldehyde-3-P: 3PG; xylulose-5-P: X5P; fructose-6-P: F6P; erythrose-4-P: E4P; glucose-6-P: G6P; and 6-phospho-gluconate: 6PG.

The draft genome of strain M82_1 has an incomplete Embden–Meyerhof–Parnas pathway (EMP pathway) because no gene encoding pyruvate kinase (phosphoenolpyruvate to pyruvate) was identified (Fig. 3, Table S6). The pentose phosphate pathway has oxidative and non-oxidative arms, and genes encoding each of these enzymes in the non-oxidative pentose phosphate pathway were present, while genes for the oxidative branch were not identified (Fig. 3, Table S6). The M82_1 draft genome also possesses an incomplete tricarboxylic acid cycle: genes encoding citrate synthase, succinate-CoA ligase, and succinate dehydrogenase were not detected (Fig. 3, Table S6). However, several genes encoding complementary anaplerotic reactions were identified (Table S6), such as aspartate ammonia-lyase (900_64), adenylosuccinate lyase (217_24), and argininosuccinate lyase (217_10), corresponding to fumarate generation, and glutamate dehydrogenase (83_28) and aspartate oxidase (217_23) for α-ketoglutarate generation. Succinyl-CoA may be supplied during the degradation of odd-chain fatty acids with the help of methylmalonyl-CoA mutase (201_6:7 and 7_7:8, Table S5). Oxaloacetate may be generated from aspartate transaminase (83_30), pyruvate carboxylase (3_34 and 65_10), pyruvate orthophosphate dikinase (1069_50), and phosphoenolpyruvate carboxykinase (1069_49, Table S5). Pyruvate has the greatest physiological importance in anaplerotic reactions, and is also the most likely to be associated with the conversion of acetyl-CoA by pyruvate ferredoxin oxidoreductase (391_25:28) and formate C-acetyltransferase (45_34, 70_81, 839_13, Table S5) (37, 79). Pyruvate may be consumed by many syntrophic bacteria under pure culture conditions (48, 82). However, genes encoding pyruvate permease were not detected in the M82_1 genome, and attempts to cultivate strain M82_1 with pyruvate failed (data not shown).

### Alkane metabolism

*Smithella* spp. are known to degrade alkanes in cooperation with methanogens under strictly anoxic conditions (13, 30, 92). Recent studies on methanogenic alkane degradation (24, 80, 81, 87) demonstrated that alkanes appear to be activated by the glycyl radical enzyme, alkylsuccinate synthase (ASS), which catalyzes the radical addition of the subterminal carbon to the double bond of fumarate, forming methyl alkyl succinate. Genes (109_1 and 226_1) encoding candidate enzymes for the anaerobic activation of *n*-alkanes in the genome of M82_1 were also detected (Fig. 3, Table S7), and they shared nearly identical sequence similarity to the putative *ass*A gene retrieved from the methanogenic hexadecane-degrading culture (14).

The primary intermediate may undergo carbon-skeleton rearrangement and decarboxylation, which were catalyzed by methylmalonyl-CoA mutase (encoded by gene 7_7:8 and 201_6:7) and carboxyl transferase (65_11), respectively. This degradation mechanism may be similar to that by which *Desulfatibacillum alkenivorans* AK-01 (11) denitrifies bacterium strain HxN1 (89).

**Fig. 4:**
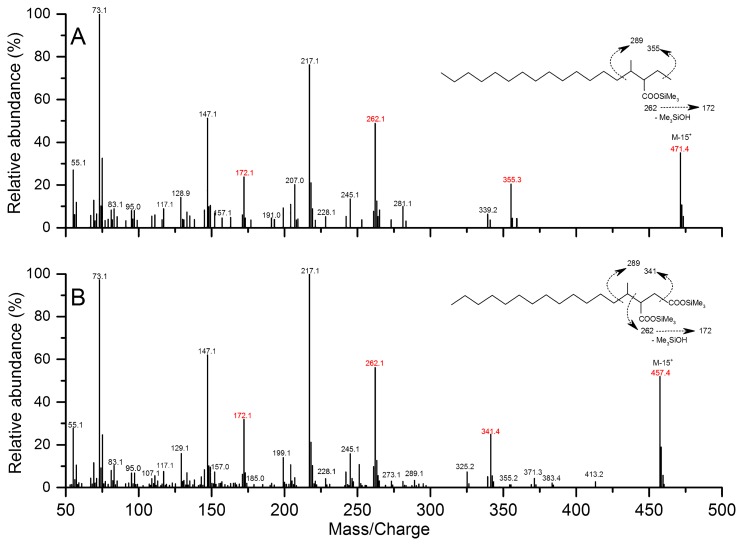
Mass spectra of silylated putative metabolites, methyl pentadecyl succinic acid (MPA) (A) and methyl tetradecyl succinic acid (MTA) (B), from cultures incubated with hexadecane and pentadecane, respectively.

The analysis of metabolites confirmed the proposed alkane degradation. Cultures amended with pentadecane and hexadecane were collected for gDNA extraction after a 298-d incubation (Fig. S3), and the T-RFLP analysis revealed that archaeal and bacterial community compositions were both similar between the pentadecane- and hexadecane-degrading cultures, albeit at slightly different relative abundances (Fig. S4). In the cultures grown with hexadecane, the M^+^ and (M-15)^+^ ions of silylated methyl pentadecyl succinic acid (MPA) metabolites occurred at m/z 471 and m/z 355, respectively. Other major ions were observed at m/z 262, 217, 172, 147, and 73 (Fig. 4A). The ion at m/z 262 was selected as a representative fragment of TMS-derivatized alkylsuccinates during the GC-MS analysis because it represents the succinyl moiety, which is generated through a McLafferty rearrangement (8, 16, 27). Further fragmentation of the signal at m/z 262 produced the ion at m/z 172 (8, 39). The ion at m/z 217 indicates trimethylsilyl transfer between carboxyl moieties, whereas the ion at m/z 147 may arise from interactions between the two functionalities (8, 63). The ion at m/z 73 may have been produced from the trimethylsilyl group in BSTFA-derivatized metabolites. Similarly, another potential metabolite representing silylated methyl tetradecyl succinic acid (MTA) was observed in the pentadecane cultures, which had an (M-15)^+^ ion at m/z 457. Other key ions included 341, 262, 172, 147, and 73, which were produced by pathways similar to those described for MPA (Fig. 4B). However, the subsequent degradation intermediates of MPA (*e.g.*, 4-methyloctadecanoic acid and 4-methyloctadec-2,3-enoic acid) and MTA (*e.g.*, 4-methylheptadecanoic acid, 4-methylheptadec-2,3-enoic acid, and 2-methylpentadecanoic acid) were not detected in the hexadecane or pentadecane culture, respectively (data not shown). Metabolites that had molecular ions at m/z 262, 457, and 471 were not detected in the control culture with similar retention times to those amended with hexadecane and pentadecane, respectively (Fig S5).

### Fatty acid metabolism

All genes required for the β-oxidation of long-chain fatty acids were identified, in which multiple ORFs encoding acyl-CoA dehydrogenase (16) and enoyl-CoA hydratase (12) were also detected (Fig. 3, Table S7), and this is consistent with our previous findings (19). Incubation experiments indicated that the M82 culture obtained energy for growth by the addition of hexadecanoic acid, dodecanedioic acid, and tetradecanedioic acid (19). A T-RFLP analysis suggested that the uncultured syntrophic hexadecane degrader survived with long-chain dicarboxylic acids (19). A genomic analysis of strain M82_1 also revealed the degradation potential of short-chain fatty acids, such as butyrate (Table S7). M82_1 contains one butyrate kinase gene, eight acetyl-CoA acetyltransferase genes, four butyryl-CoA dehydrogenase genes, twelve enoyl-CoA hydratase genes, two 3-hydroxybutyryl-CoA dehydrogenase genes, and two phosphate butyryltransferase genes (Table S6). The β-oxidation of even- and odd-numbered fatty acids produces acetyl-CoA and propionyl-CoA, respectively. Two propionate-oxidizing pathways via methylmalonyl-CoA (38, 65) and a six-carbon intermediate metabolite (18, 45) have been proposed in syntrophic bacteria. The absence of several key coding genes (*e.g.*, succinate dehydrogenase) in the methylmalonyl-CoA pathway in the binning genome indicated that M82_1 is incapable of propionate oxidation (Table S7). The second pathway has been proposed for *S. propionica* (18, 45), which shared high sequence similarity (96% 16S rRNA gene sequence identity) with this M82_1 strain (13). However, there is currently no information regarding the genetics of syntrophic propionate oxidation, which precluded a systematic comparative analysis. Moreover, no obvious growth of syntrophic alkane degraders was observed in a sub-culture of M82 with propionate as the sole substrate (19).

### Nitrogen and sulfur metabolism

Strain M82_1 appears to be able to source ammonia directly from the environment using specific transporters (231_3, 450_3, and 451_1, Fig. 3). The draft genome contains a common core of nitrogen fixation *nif* genes (*nif*HDKEB) clustered in the scaffold of 227 (227_17:23), which encode the structural subunits of molybdenum-dependent nitrogenase (Table S8). It has been proposed that nearly all diazotrophs have a minimal gene set consisting of six conserved genes: *nif*H, *nif*D, *nif*K, *nif*E, *nif*N, and *nif*B (21). However, there is evidence of nitrogen fixation in species lacking *nif*N (49). The potential function of nitrogen fixation was proposed to tolerate acidification and provide hydrogen and ammonia for partner hydrogenotrophic methanogens (52), which has attracted increasing attention in syntrophic hydrocarbon-degrading species. The draft genome also contained genes (437_38, 83_20, 22, 23, 26, and 28) that encode the glutamine synthetase/glutamate synthetase (GS/GOGAT) and glutamine dehydrogenase (GDH) pathways (Fig. 3, Table S8). Both pathways contribute to the assimilation of ammonia into organic nitrogen compounds, which may be used as intracellular metabolites (12).

Genes responsible for reducing inorganic sulfur species, such as sulfate, elemental sulfur, thiosulfate, and sulfite, were not detected. Only genes (48_57, 155_29:30) for the conversion of sulfide to cysteine were detected in the M82_1 genome, suggesting the fixation of inorganic sulfide into a carbon skeleton (Table S8). However, culture M82 performed the methanogenic degradation of hexadecane with concurrent sulfate reduction in a transfer culture incubation amended with sulfate, and a decrease in methane production with an increased initial sulfate concentration was also observed (Ma *et al.*, in preparation). This result indicates that some sulfatereducing bacteria, rather than the syntrophic alkane degrader M82_1, possess the capacity to perform anaerobic respiration using sulfate as a terminal electron acceptor.

### Energy conservation and electron flow

Genomic analyses revealed that electron-accepting systems using oxygen, nitrate, and metal respiration were not present in the M82_1 genome. Energy-conserving mechanisms in anaerobic life may be divided into three modes: substrate-level phosphorylation (SLP), electron transport phosphorylation (ETP), and flavin-based electron bifurcation (FBEB) (7). Acetyl-CoA is a high-energy compound and a central molecule in the metabolism of syntrophic bacteria. One gene (108_4) was found to encode AMP-forming acetyl-CoA synthetase (Table S9) and shares 68% sequence similarity to SYN_02635 of *S. aciditrophicus*, which has been proposed to synthesize ATP from acetyl-CoA (84) In addition, M82_1 appears to fuel its ATP synthesis through proton translocation based on the detection of an F_1_F_0_-type ATP synthase in the genome, in which the cytoplasmic F_1_ domain is encoded by 463_2:7, and the membrane-integral F_0_ domain is encoded by dispersed genes (437_33, 463_8:9, 4_23, and 31; Table S9). A membrane-bound sodium-translocating pyrophosphatase (PPase) encoded by gene 403_42 couples proton translocation across the cytoplasmic membrane, which may be associated with energy conservation via ATP synthase (Fig. 3, Table S9). A similar proton-translocating pyrophosphatase has been reported in syntrophic benzoate-degrading *S. gentianae*, in which pyrophosphate hydrolysis by the membrane-bound pyrophosphatase was associated with proton translocation across the cytoplasmic membrane, and the ratio of ATP formation/pyrophosphate hydrolysis was 1:3 (70). The oxalate/formate antiporter in anaerobic *Oxalobacter* species catalyzes the exchange of extracellular oxalate, a divalent anion, and intracellular formate, the monovalent product of oxalate decarboxylation, which results in an internally negative membrane potential and generates the proton motive force necessary for ATP production. This antiporter was proposed as a “virtual proton pump” (35). The detection of genes 889_8 and 900_72 encoding oxalate/formate antiporters suggests that strain M82_1 also constitutes an “indirect” proton pump, the operation of which may sustain the proton-motive force characteristic of bacterial systems (Fig. 3, Table S9). In addition, the cellular concentrations of protons and sodium ions may be regulated by sodium/proton antiporters encoded by ORFs 25_8 and 900_33. These findings suggest a critical role for protons and sodium in the bioenergetics of “*Candidatus* Smithella cisternae” strain M82_1.

H_2_ and formate are electron sinks in syntrophic bacteria (77). Genomic analyses predicted the presence of four formate dehydrogenases (FDHs) and four hydrogenases (HYDs) in the *Candidatus* Smithella cisternae (Table S10). Among them, two FDHs (encoded by 7_13:15 and 69_12:15) contained Tat motifs (Fig. 3, Table S10), indicating that the corresponding proteins are translocated to the periplasm upon maturation (55). Furthermore, domain “IPR006443” was exclusively present in the extra-cytoplasmic FDH alpha subunit, which represents a typical difference between syntrophic and non-syntrophic butyrate and propionate degraders (91). The detection of domain “IPR006443” in both FDHs suggests that strain M82_1 has the ability to syntrophically degrade short-chain fatty acids (Fig. 3, Table S10). Gene clusters 593_2:3 and 69_3:4 likely encode the iron-sulfur and alpha subunits of a cytoplasmic FDH, respectively. Gene clusters 900_60:63 and 391_22:24 are predicted to encode cytoplasmic [FeFe]-HYD components (Fig. 3, Table S10). A comparison of these cytoplasmic FDH- and [FeFe]-HYD-encoding genes with those of other syntrophic fatty acid-degrading bacteria (*i.e.*, *S. fumaroxidans*) revealed a high degree of homology at the amino acid level (Table S10). Genomic and biochemical analyses suggested that HYD1 (Sfum_0844:46) and FDH1 (Sfum_2703:07) of *S. fumaroxidans* likely confurcate electrons from NADH and ferredoxin (Fd) to protons and carbon dioxide to produce H_2_ and formate, respectively (61, 90). A similar mechanism has been proposed for H_2_ generation in *Thermotoga maritima* (71). Sequence similarities implied that M82_1 employs electron confurcation for formate and H_2_ production from NADH, as previously proposed (51).

Fd plays a central role in the energy metabolism of many anaerobic bacteria and archaea and it evolved during the very early stages of evolution (23, 78). Like many other syntrophic bacteria (53, 54, 74), the M82_1 genome does not encode the Rnf complex necessary for the reduction of Fd from NADH. A potential source of reduced ferredoxin (Fd_red_) may be generated by heterodisulfide reductase/methyl-viologen-reducing hydrogenase (HdrABC/MvhD)-like compounds, encoded by a gene cluster (437_38:51), which was named Hdr/Flox proteins in syntrophic bacteria (52). The HdrABC/MvhADG complex in methanogens couples the unfavorable reduction of Fd to the favorable reduction of CoM-S-S-CoB heterodisulfide with electrons derived from H_2_ or formate (83). Similarly, Hdr/Flox gene clusters were also found in anaerobic bacteria including syntrophic fatty acid-degrading bacteria (52, 53), sulfate-reducing bacteria (58), acetogens (60), and syntrophic alkane-degrading *Smithella* (24, 79, 80, 87), which has been proposed to play a role in flavin-based electron confurcation with an uncharacterized thiol–disulfide redox pair (52, 53). (Fig. 3). However, further biochemical research on the complex is needed in order to obtain more detailed information.

The M82_1 genome has two gene clusters (108_2:3 and 155_20:21) that encode ETFs (Fig. 3, Table S10). One set of ETF genes (108_2:3) shared a high degree of homology at the amino acid level (>78% and >44%, respectively) with their respective counterparts in *S. aciditrophicus* (SYN_02637:6) and *S. wolfei* (Swol_0697:6). This set of ETFs was adjacent to a gene (108_1) predicted to encode the transmembrane FeS-binding oxidoreductase “DUF224” with unknown function, 73% sequence identity to SYN_02638, and 45% homology to Swol_0698. In *S. wolfei*, the Bcd/EtfAB/DUF224 complex is presumed to link electron flow from butyryl-CoA dehydrogenase to the membrane. The co-expression of the gene cluster (Swol_0696:8) confirmed that electrons derived from butyryl-CoA are transferred through a membrane-bound EtfAB:quinone oxidoreductase (DUF224) to a menaquinone cycle and further via a *b*-type cytochrome to an externally oriented formate dehydrogenase (68, 75).

### The CRISPR/Cas system

The M82_1 genome has five CRISPR regions distributed in scaffolds 437, 536, and 592 (Table S11). The three 10-, 20-, and 77-spacer CRISPR repeat sequences located in scaffold 437 shared 100% homology with the violet-pigmented bacterium *Chromobacterium violaceum* ATCC 12472 (CRISPR id: NC_005085_1). The 23-spacer CRISPR repeat sequence in scaffold 536 showed 95% sequence similarity with the hyperthermophilic archaeum *Thermoproteus tenax* Kra 1 (CRISPR id: NC_016070_6). The 25-spacer exhibited 86% homology with the piezophile *Photobacterium profundum* SS9 (CRISPR id: NC_005871_1). The M82_1 genome also harbored two gene clusters encoding *cas* proteins: one (536_53:54) is related to a type V CRISPR-associated endonuclease, and the other (519_19:21) encodes proteins of the Repeat-Associated Mysterious Proteins (RAMP) superfamily.

### ABC transporters

ATP-binding cassette (ABC) transporters are an important class of transport proteins that use the energy derived from the hydrolysis of ATP to ADP, are widely distributed in all domains of life, and are involved in a large variety of processes (69). Genes encoding metal ion transport systems for molybdate, cobalt, nickel, zinc, tungsten, and ferric iron were detected in the M82_1 genome (Table S12), indicating that metal acquisition is essential for anaerobic metabolism. The presence of ABC transporters related to organic solvent efflux (227_5:7) suggested that strain M82_1 thrives under the adverse conditions imposed by toxic chemicals (47). The identification of transporters for oligopeptides, lipoproteins, and lipopolysaccharides in the M82_1 genome requires further study because, based on the genomic analysis, M82_1 possesses a limited carbohydrate metabolic capability (Fig. 3).

## Conclusion

Members of the *Syntrophaceae* phylotype represent some of the most abundant syntrophic alkane-degrading bacteria and appear to play an important role in the carbon cycle in hydrocarbon-contaminated environments (20). In the present study, the combination of metagenomics and GC-MS successfully elucidated the genetic and metabolic properties of a *Syntrophaceae* sublineage II bacterium, which was proposed as the novel species “*Candidatus* Smithella cisternae”. Alkane activation was predicted to be accomplished by fumarate addition under methanogenic conditions. Genes encoding members of the β-oxidation pathway for the degradation of alkane-degrading intermediates (including butyrate) to acetate were revealed. ATP was synthesized by substrate-level phosphorylation from acetyl-CoA or F_1_F_0_-ATP synthases. Moreover, strain M82_1 may employ electron-confurcating FDH and HYD or ETF and transmembrane FeS-binding oxidoreductase to overcome the energy barrier during the production of H_2_/formate using electrons derived from NADH or β-oxidation. In addition, an Hdr/Mvh-like complex, instead of an Rnf complex, was proposed to produce Fd_red_. The *Candidatus* Smithella cisternae strain M82_1 genome provides another genetic reference to deepen our understanding of the diverse ecological functions of this important genus and sheds light on its applications in alkane degradation in hydrocarbon-contaminated environments.

### Taxonomic proposal for “*Candidatus* Smithella cisternae”

We propose the provisional taxonomic assignment of sublineage II of the Family *Syntrophaceae* (20) as “*Candidatus* Smithella cisternae” because of the absence of a pure culture. Smithella cisternae refers to the isolation of the organism from an oil field (cisternae) (cis.ter’na.e. L. gen. n. cisternae, of a subterranean reservoir, enriched from an oil field).

## Supplementary Material





## Figures and Tables

**Table 1:** General features of the assembled metagenome and binned M82_1 genome.

Items	Binned (M82)
Contigs (no.)	236
DNA (bp)	2,753,163
Min. sequence length (bp)	506
Max. sequence length (bp)	111,138
N_50_ length (bp)	35,699
G+C content (%)	42.8
ORFs (no.)	2,815
ORFs with an assigned function	2,764
ORFs with KEGG matches	1,184 (42%)
ORFs with COG matches	1,937 (69%)
ORFs with GO matches	1,267 (45%)
ORFs with Pfam matches	2,232 (79%)
Number of copies of the rRNA operon	1
CRISPR	5
ORFs with CAZYme matches	108
