# Supplementary material for: Metagenomic Characterization of Candidatus Smithella cisternae Strain M82_1, a Syntrophic Alkane-Degrading Bacteria, Enriched from the Shengli Oil Field
Source: Microbes Environ. 2017 Sep 27;32(3):234–43. doi: 10.1264/jsme2.ME17022 (PMC5606693; doi:10.1264/jsme2.ME17022)
Supplement: Supplementary file 2 [file 32_234_s2.pdf]

Fig. S1A   Qin et al.,

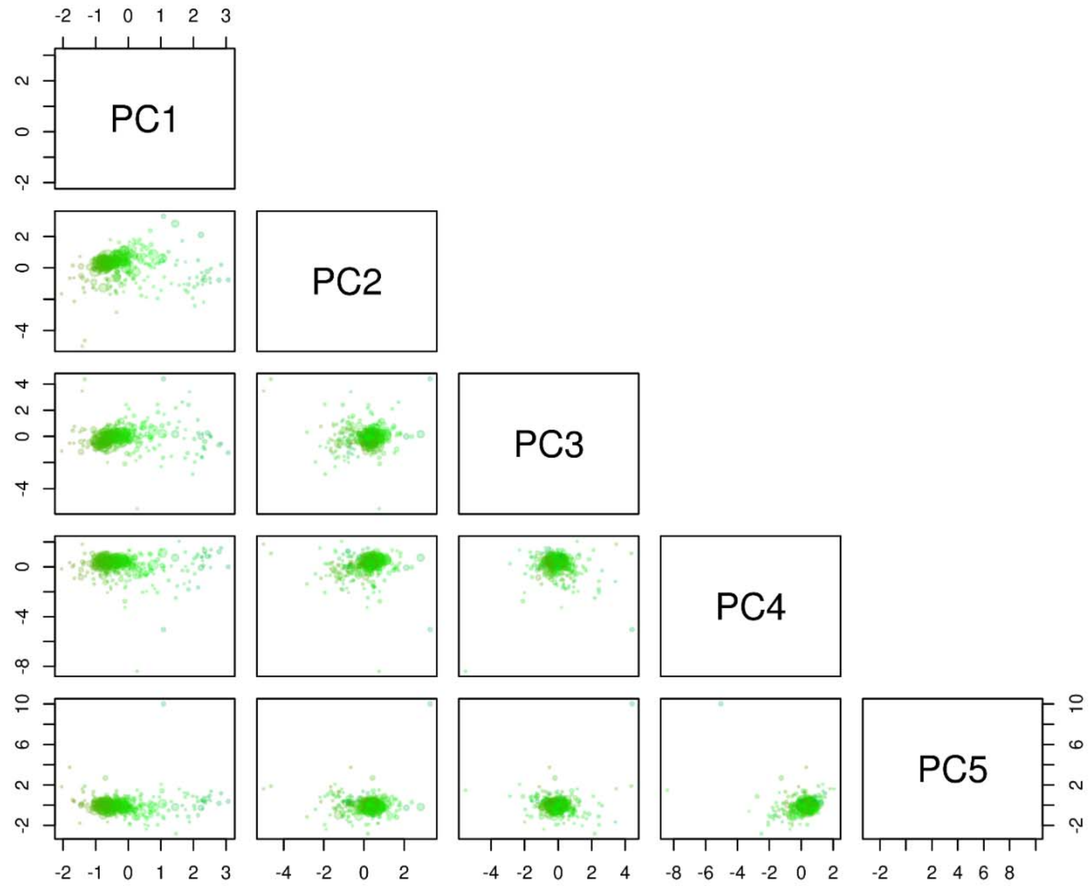

Fig. S1 PCA of the TNF of the binned genome before (A) and after (B) the archaeal and low quality scaffolds were excluded

Fig. S1B   Qin et al.,

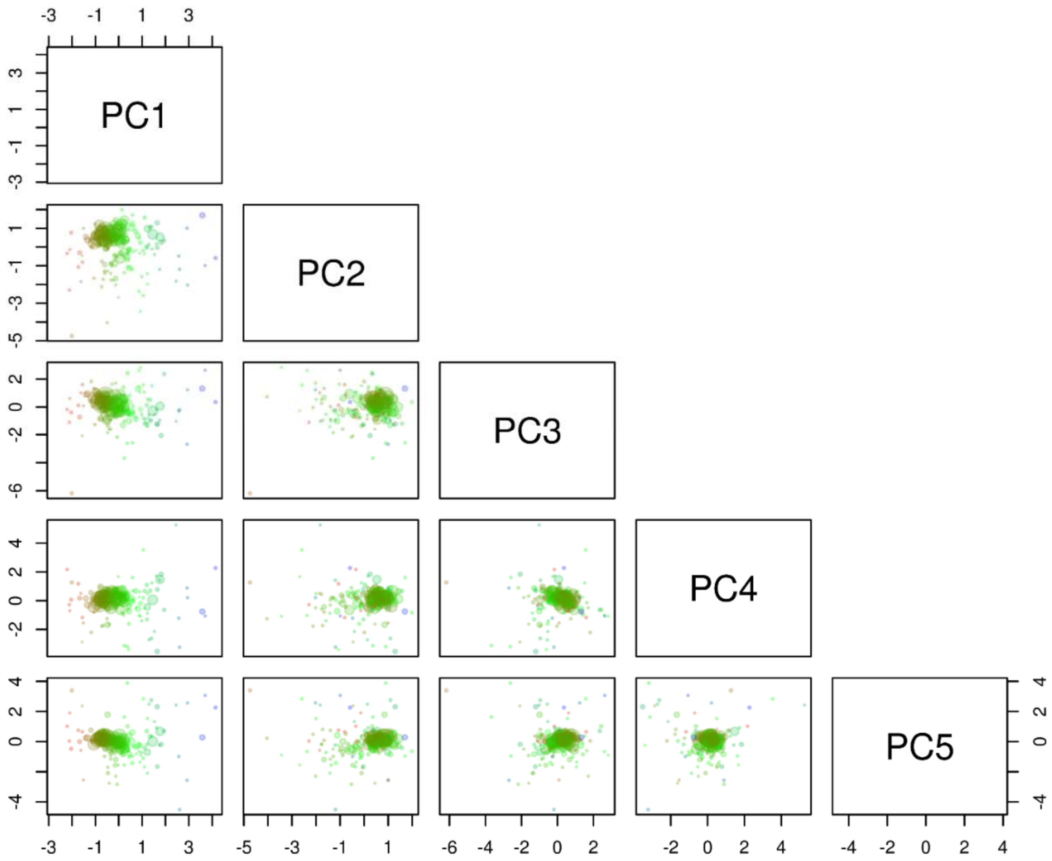

Fig. S1 PCA of the TNF of the binned genome before (A) and after (B) the archaeal and low quality scaffolds were excluded

Fig. S2 Qin et al.,

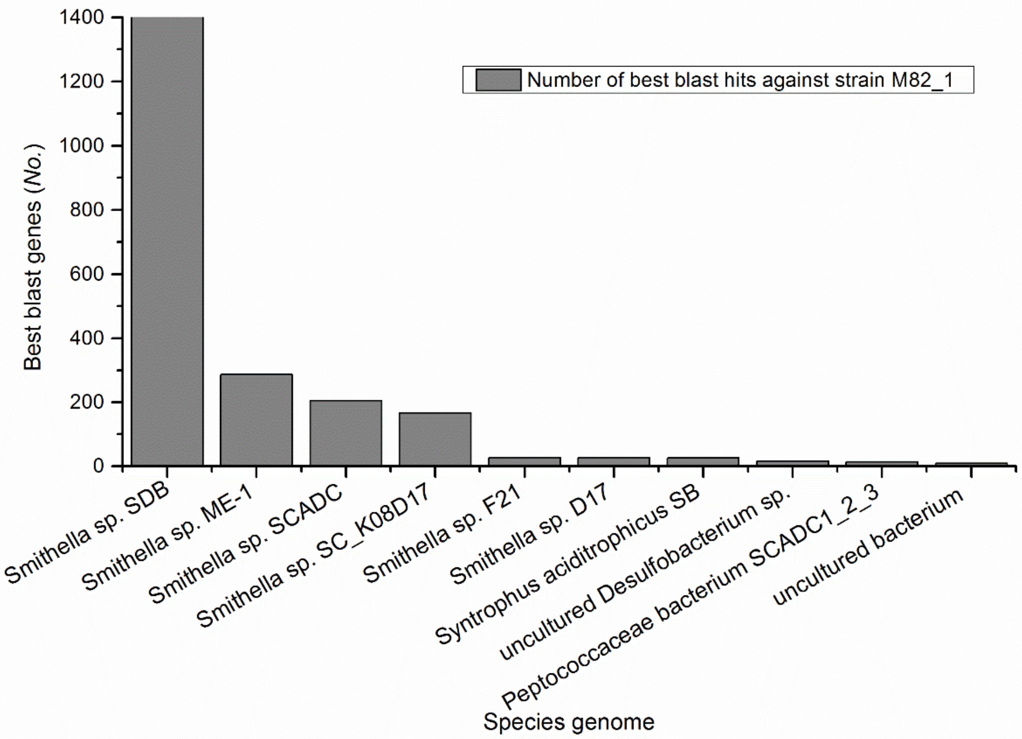

Fig. S2 Best BLAST hit distribution of "Candidatus *S. cisternae* strain M82_1" ORFs with other genomes

Fig S3 Qin *et al*.,

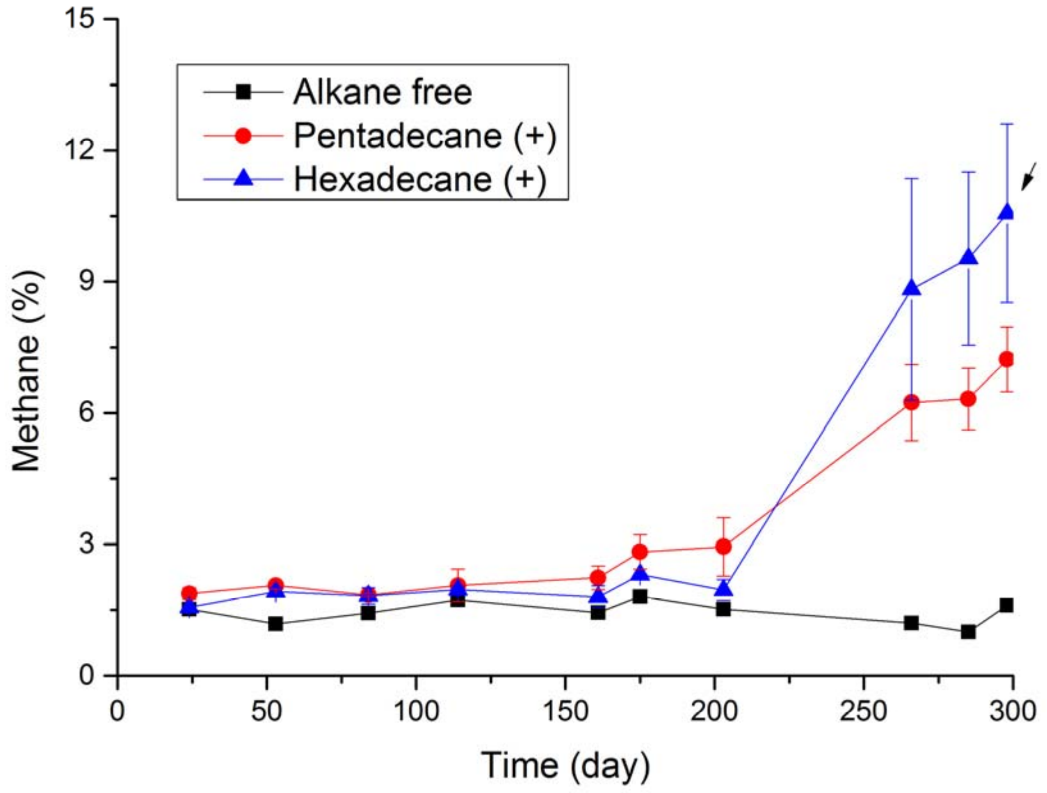

Fig. S3 Methane production from hexadecane and pentadecane addition, respectively

Fig. S4 Qin *et al*.,

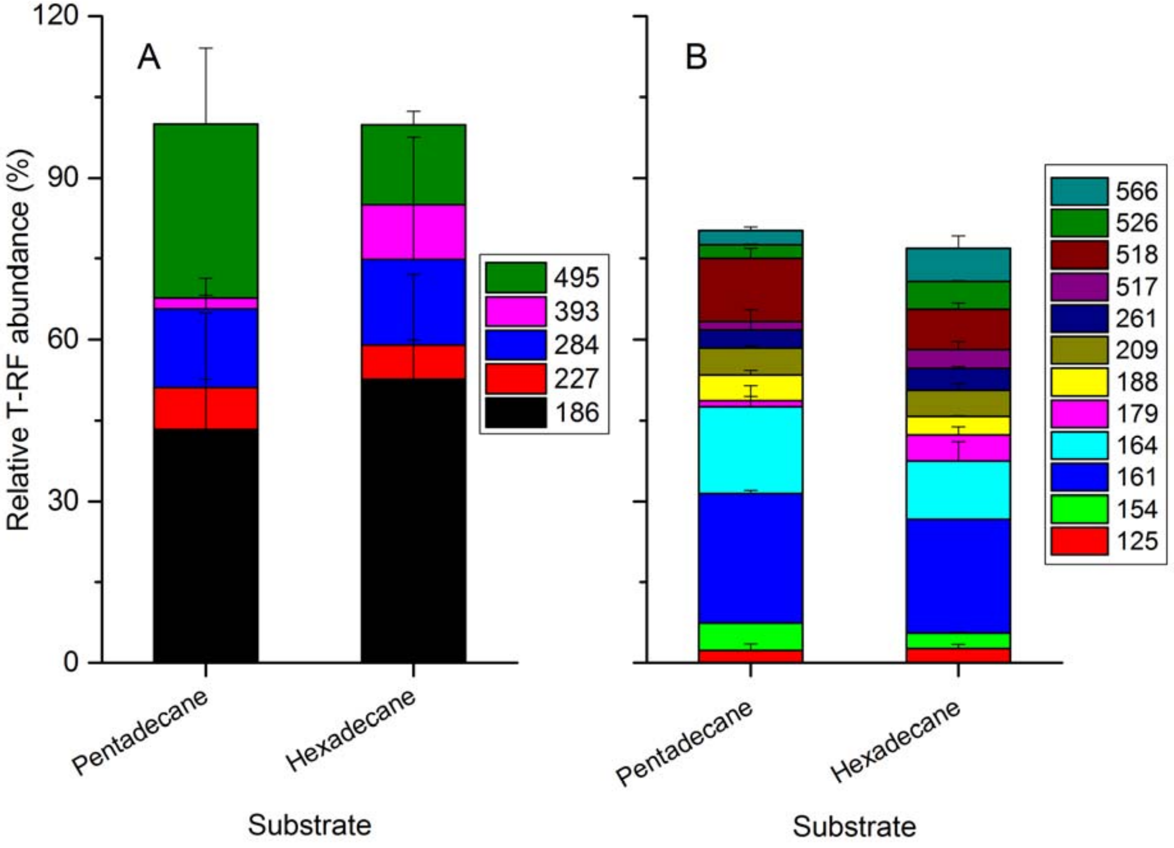

Fig S4. Archaeal (A) and bacterial (B) diversity of methanogenic cultures amended with pentadecane and hexadecane after 298 days of incubation.

Fig. S5 Qin et al.,

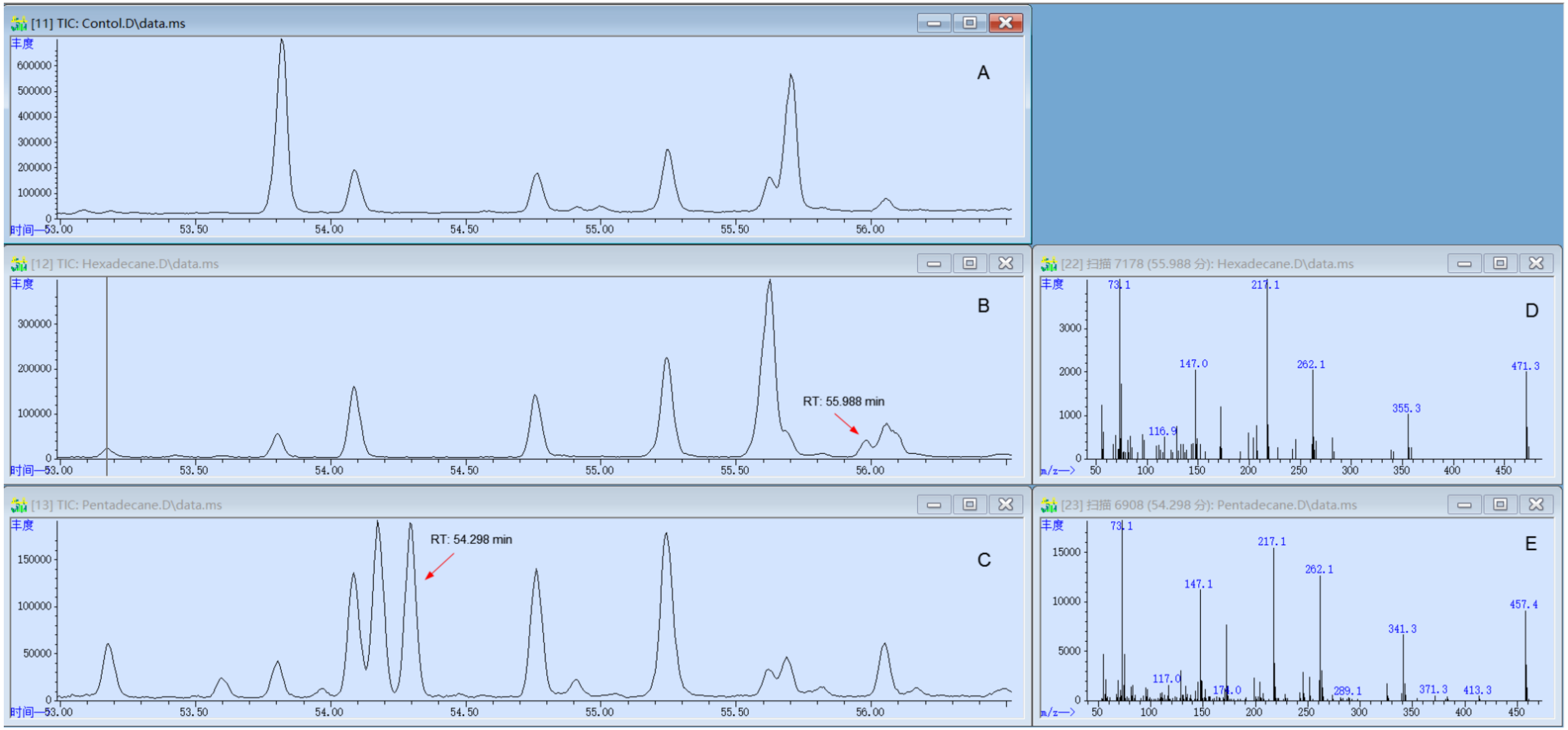

Fig. S5 Gas chromatograms of fatty acid profiles of cultures M82 grown without long chain alkanes (A), with hexadecane (B) and pentadecane (C), and mass spectra of the silylated putative metabolites methylpentadecysuccinic acid (MPA) (D) and methyltetradecysuccinic acid (MTA) (E) from cultures incubated with hexadecane and pentadecane, respectively.
